# Genetic Signature of Human Pancreatic Cancer and Personalized Targeting

**DOI:** 10.3390/cells13070602

**Published:** 2024-03-29

**Authors:** Stephan J. Reshkin, Rosa Angela Cardone, Tomas Koltai

**Affiliations:** 1 Department of Biosciences, Biotechnologies and Environment, University of Bari “Aldo Moro”, 70125 Bari, Italy; stephanjoel.reshkin@uniba.it; 2 Oncomed, Via Pier Capponi 6, 50132 Florence, Italy

**Keywords:** PDAC (pancreatic ductal adenocarcinoma), driver mutations, KRAS, personalized treatment

## Abstract

Pancreatic cancer is a highly lethal disease with a 5-year survival rate of around 11–12%. Surgery, being the treatment of choice, is only possible in 20% of symptomatic patients. The main reason is that when it becomes symptomatic, IT IS the tumor is usually locally advanced and/or has metastasized to distant organs; thus, early diagnosis is infrequent. The lack of specific early symptoms is an important cause of late diagnosis. Unfortunately, diagnostic tumor markers become positive at a late stage, and there is a lack of early-stage markers. Surgical and non-surgical cases are treated with neoadjuvant and/or adjuvant chemotherapy, and the results are usually poor. However, personalized targeted therapy directed against tumor drivers may improve this situation. Until recently, many pancreatic tumor driver genes/proteins were considered untargetable. Chemical and physical characteristics of mutated KRAS are a formidable challenge to overcome. This situation is slowly changing. For the first time, there are candidate drugs that can target the main driver gene of pancreatic cancer: KRAS. Indeed, KRAS inhibition has been clinically achieved in lung cancer and, at the pre-clinical level, in pancreatic cancer as well. This will probably change the very poor outlook for this disease. This paper reviews the genetic characteristics of sporadic and hereditary predisposition to pancreatic cancer and the possibilities of a personalized treatment according to the genetic signature.

## 1. Introduction

Most solid cancers are the consequence of a multistep process of genetic and epigenetic changes that transforms normal cells into cancer cells. This does not seem to be the case for many hematological tumors where the tumor is driven by a single gene. On the other hand, pancreatic cancers are the result of multiple genetic alterations that function through a relatively small number of pathways [1].

Tumors born from different tissues tend to have a different set of mutations that are generally characteristic for each cancer. For example, Braf mutations such as V600E are one of the most frequently found mutations in malignant melanoma. In this mutation, a valine residue (V) is substituted by glutamic acid (E) in position 600 of the Braf protein. This does not mean that Braf V600E can only be found in melanoma, because it has also been found in cholangiocarcinoma, sarcoma, glioma, neuroendocrine carcinoma, and salivary gland carcinomas [2], but it is quite infrequent in non-melanoma tumors. Pancreatic adenocarcinoma has its “own” set of mutated gene signatures that, although non-exclusive, are found very frequently.

There is evidence that until a symptomatic pancreatic adenocarcinoma (PDAC) develops, it takes around fifteen years from when the first pro-tumor genotypic change occurs [3]. The growth rate is not particularly faster than those of other tumors [4]. Without entering into the debate of which is the tumor-originating cell [5] (acinar, ductal, or both), PDAC development follows a step-by-step process from intraepithelial dysplasia, to intraductal neoplasia, to full-blown invasive adenocarcinoma [6,7]. There is a series of precursor lesions that can generate the invasive PDAC, such as intraepithelial neoplasia, intraductal papillary mucinous tumors [8] and mucinous cystic neoplasm. While the first is microscopic, the other two are macroscopic [9]. Intraepithelial neoplasia (PanIn) is the most frequently found PDAC precursor lesion [10].

**Figure 1:**
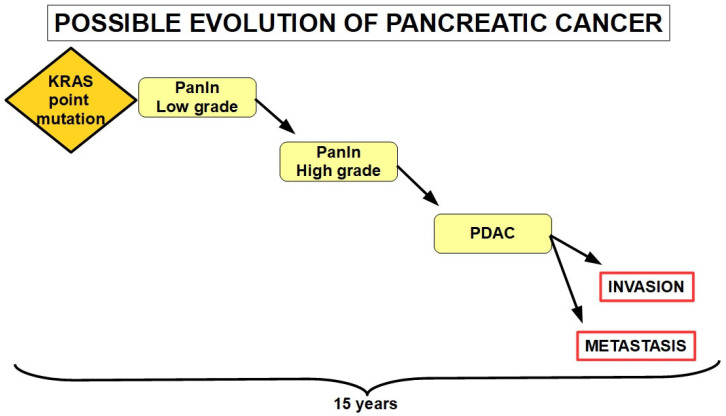
This is the most frequent evolution scheme of PDAC, which takes approximately 15 years to become symptomatic since the first pro-tumoral mutation occurs. In this figure, KRAS point mutation is the initiator of the tumor [13]. For this progression to take place, specific genes need to be mutated or epigenetically silenced. Since 1988, the association of KRAS mutation with pancreatic cancer has been well established [14]. However, there is another 10 or 15% of the cases (KRAS wild type tumors) in which a different driver gene must be playing a role.

A growth model for pancreatic cancer progression can be built following the multi-step scheme that was originally proposed by Vogelstein, Feron, and Kinslay for colorectal carcinoma [11,12] (Figure 1).

It has been believed for years that the initial lesion in the pancreas is a mutation of an intraductal cell, the founder mutation, leading to intraductal proliferation or intraductal hyperplasia and to the term “ductal adenocarcinoma” [15]. However, an acinar or even centroacinar origin cannot be disregarded [16]. It is this dysplastic duct or acinar cell that, through clonal evolution, evolves into the specific molecular subtypes of the invasive adenocarcinoma with different aggressiveness and prognosis profiles [17,18]. These findings have been corroborated on clinical grounds [19,20] and by genetic research [21,22,23,24]. For example, KRAS mutation, the most frequently found driver mutation of pancreatic cancer, has also been frequently found in intraductal carcinoma [25,26]. Other shared genetic alterations are BRCA2 mutations and loss of p53 and p16 [27,28,29,30]. These mutations accumulate over time (an estimated 15 years), and this is the possible reason why sporadic PDAC appears mainly in patients over 60 years of age, while it presents in a younger population with hereditary cancer-predisposing syndromes [21,22,23,24,25,26,27,28,29,30,31,32,33]. In this last case, the existent hereditary mutation seems to decrease the time required for clonal evolution. Sporadic PDAC in patients under 55 represent less than 10% of cases [34].

There are essentially two different tumors that can arise in the pancreas: endocrine and exocrine tumors. This review will be focused on cancers originating in the exocrine pancreas where 90% of cases are pancreatic ductal adenocarcinomas (PDAC) [35,36].

In spite of considerable improvements in cancer therapy in general, exocrine pancreatic cancer remains a highly fatal disease which is quite difficult to treat. Five-year survival is less than 5% in non-operable cases and around 20% in surgical cases [37,38,39].

Pancreatic cancer is becoming a public health problem because the number of cases is constantly increasing [40], and treatment results are quite poor [41,42,43]. Incidence is growing between 0.5 and 1% each year [44]. In 1985, pancreatic cancer was the eighth most frequent cause of cancer mortality [45]. After only thirty years, it was the fourth [46], and it has been forecast that it will be the second most common by 2030–2040 [47,48]. There is no clear explanation for this increase. The incidence of pancreatic cancer is higher in developed countries [49]. While longer average lifespan in developed countries may have some influence in this regard, there are probably other environmental and clinical circumstances (diet, alcohol, obesity, diabetes, chronic pancreatitis, smoking, Helicobacter pilori infection and gut microbiota) that cooperate to determine this higher incidence [50].

While surgery is the best option (and treatment of choice), less than 20% of patients are candidates for a surgical approach [51,52,53]. This is a consequence of the usual late diagnosis [54]. Tumor stage at the time of diagnosis is the single most important factor that determines outcome [55]. Symptomatic patients are usually incurable [56]. Screening methods have not yet shown to be effective for early detection of the disease [57,58,59].

Generally, resectable cases have a 5-year survival of 20–30% [60,61]. However, in tumors that are less than 1 cm, the five-year survival increases to approximately 60% [62,63], underlying the absolute importance of early diagnosis.

Neoadjuvant chemotherapy or chemoradiotherapy [64,65] (although with some controversies [66]) and better surgical techniques [67,68] have slightly increased the proportion of operable cases, and this has produced a minor increase in overall survival in the last decade. However, there has been no significant survival increase in non-surgical patients.

Most pancreatic cancers are sporadic, but there is a small group of patients related to hereditary cancer-predisposing syndromes such as BRCA1/2 mutation, familial pancreatic cancer, Peutz-Jeghers, Lynch, Li-Fraumeni syndromes and familial atypical multiple mole melanoma [69].

Non-hereditary risk factors such as chronic pancreatitis, diabetes and smoking have also been identified [70]. Some of these factors are preventable, such as smoking, but most of them are not.

The standard chemotherapeutic approach consists of gemcitabine associated with nanoparticle albumin-bound paclitaxel (nab-paclitaxel) or the FOLFIRINOX scheme (oxaliplatin, irinotecan, fluorouracil and leucovorin) and associated radiotherapy. However, chemoradiotherapy offers poor results regarding patient survival in advanced tumors [71], although some minor improvements have been reported recently [72].

A genetically personalized approach will probably improve overall survival. This means that genotyping pancreatic cancer is becoming a necessary tool for an adequate treatment. Furthermore, specific drugs have been developed against many PDAC driver genes and driver pathways, as discussed below.

## 2. Molecular Characteristics of Sporadic PDAC

When examining the microarray of genes whose expression is modified in pancreatic cancer, Grutzmann et al. [73] found that 568 genes had a “consistently and significantly” altered expression (364 were up-regulated, and 204 were down-regulated). This does not mean that all of these genes are essential for transformation and malignant evolution. Many of them belong to those considered passenger genes (non-essential for transformation and evolution) as opposed to the concept of driver genes (essential for transformation and/or evolution) [74]. Furthermore, all tumors, including PDAC, show an accumulation of driver and passenger mutations during their evolution [75].

Pancreatic cancer, like all other cancers, is a genetic disease and has specific mutated genes. These include tumor suppressor genes and oncogenes.

Gene mutations can follow a step-by-step process during the development and progression of PDAC.

Initially, small tumors and pre-invasive lesions have mutations of KRAS, Muc5 and Her2 (early mutations). Intermediate lesions, usually less than 2 cm in diameter, are also mutated in the genes for p16, Muc5 and cyclin D1, while bigger lesions have further mutations in TP53, DPC4/SMAD4 and BRCA2 [76]. Despite PDAC heterogeneity at the molecular and metabolic levels, there is an emerging genetic pattern that will be considered below [77].

### 2.1. Tumor Suppressor Gene Mutations

Among tumor suppressor genes, CDKN2A/p16 (p16 inhibitor of cyclinD/Cdk-4 complexes) have been found to be inactivated by mutation in more than 90% of PDACs [78,79]. The second mutation in frequency is TP53 (60%) [80,81], followed by *MADH4/DPC4/SMAD4 (50%)* [82]. TP53 and MADH4/DPC4 are found in intraepithelial neoplasia and seem to be late mutations in progression [83]. BRCA2 and MLH1, which are DNA repair genes, were found to be mutated in approximately 7% of cases.

### 2.2. Oncogene Mutations

Considering the oncogenes, KRAS is mutated in 80–95% of PDACs [84,85] and can be considered a very important, if not the most important, driver gene.

EGFR amplification, but not mutation, is a frequent finding (40%) of PDAC [86].

In 66 Korean patients with inoperable PDAC, Lee et al. found 32 cases with a KRAS point mutation and one with EGFR mutation. A total of 26 patients showed EGFR amplification [87]. The KRAS mutation is more frequent in Western populations. Almoguera et al. found 21 patients with mutated KRAS out of 22 cases [88].

### 2.3. Driver Genes of PDAC

Four canonical driver genes have been identified in PDAC [89]:

- The Kirsten rat sarcoma (KRAS).
- Cyclin dependent kinase inhibitor 2 (CDKN2A/p16), also known as MTS1 or multiple tumor suppressor 1.
- Tumor suppressor protein 53 (TP53).
- Small Mothers Against Decapentaplegic homolog 4 (MADH4/DPC4/SMAD4).

The best-known and -studied driver gene is the pro-proliferative mutated KRAS. However, SMAD4 mutation or loss is also an important driver which increases metastatic dissemination [90]. The percentage of these mutation varies in different publications. As an example, Box 1 shows the percentage of genetic mutations published in the ‘Cosmic’ database as of December 2023.

Box 1 **PDAC mutations registered in the Cosmic database accessed 10 December 2013 (https://cancer.sanger.ac.uk/cosmic).**

COSMIC DATABASE: 12333 PDAC REGISTERED CASES, % MUTATIONS COSMIC: Catalog of somatic mutations in cancerKRAS64% (8896 samples)CAMTA120%TP53% (3111 samples)AFF318%SMAD419% (2997 samples)PREX216%LRP1B28%ALK10%ERBB417% (2175 samples)LPP16%PTPRT20%ZNF52115%ZFHX319%GRIN2A11%FHIT22%EBF115%CDKN2A9% (3108 samples)FOXP111%NTRK314%RAD51B11% (1799 samples)

The 64% KRAS mutation seems excessively low compared with other publications. Most statistics show that the KRAS mutation rate is above 90% in PDAC. Some databases are built on information obtained through conventional sequencing technologies that report a KRAS activating mutation in 70–80% of samples, while more recent data based on next generation sequencing indicate a frequency above 90% [91]. However, some of these statistics are based on selected populations that introduce a certain bias. Indeed, while Biankin et al. found 93% of mutated KRAS in 142 patients in early-stage sporadic PDAC [92], other authors have found a lower percentage of Kras mutation. For example, Luo found 85% [93], Waters and Der 84% [94], Kim et al. 83% [95], Hashimoto et al. 92.8% [96], Philip et al. 89.3% [97], and Miglio et al. 85.2% [98]. Therefore, it is reasonable to expect around 80 to 90% of the population having KRAS mutations. Interestingly, a study on the Brazilian population, which has an extensive interethnic admixture, found only 60% of mutated KRAS [99]. On the clonal evolution side, KRAS mutations were present in 36% of PanIN-1A, 44% of PanIN-1B, and 87% of high-grade PanIN lesions (PanIN-2 and PanIN-3) [100].

### 2.4. Alternative Driver Genes

When wild type KRAS is found in a pancreatic adenocarcinoma, there is always some alternative gene, usually a growth factor receptor or RAS family gene that replaces KRAS as the leading cancer instrument. Genes such as GNAS (Guanine Nucleotide binding protein, Alpha Stimulating activity polypeptide), BRAF, CTNNB1, and other RAS pathway genes were found to be driving the tumor.

#### 2.4.1. GNAS

GNAS codes for the alpha subunit of a G-protein coupled receptor. GNAS has been found to promote STAT3, which stimulates growth and invasion in hepatocellular carcinoma [101]. Interestingly, GNAS mutations seem to be involved in the production of pancreatic cysts, particularly intraductal papillary mucinous neoplasms [102,103,104].

The mechanisms involved in tumor driving in addition to STAT3 promotion seem to be the inhibition of the tumor suppressor salt-inducible kinases 1–3 (SIK 1–3) [105]. It is not infrequent to find GNAS mutations accompanying driver KRAS mutations [106].

#### 2.4.2. BRAF

BRAF gene/protein is part of the RAS/RAF/MEK/ERK pro-proliferative and pro-tumoral pathway. BRAF has been found to be a driver gene/protein in some pancreatic cancers [107]. BRAF V600E mutations (incidence 3%) [108] and deletions and fusions in the N486-P490 region can be found. Importantly, BRAF V600E-driven pancreatic cancers should be susceptible to specific BRAF inhibitors.

#### 2.4.3. CTNNB1

CTNNB1 is the gene on chromosome 3 that encodes beta catenin. CTNNB1 mutations alter the Wnt/*β*-catenin pathway, inducing transcription of genes that regulate the cell cycle. In 16 PDAC tumors examined, only two were found to have a CTNNB1 mutation, but around 65% showed increased *β*-catenin [109]. Kubota et al. [110] found that missense mutations were present in 7 out of 7 patients with solid pseudopapillary neoplasms (tumors with low malignant potential) but in none of 16 biopsies in PDAC patients.

#### 2.4.4. FGFR2

FGFR2 is the gene that codes for fibroblast growth factor receptor 2, which has been found to be mutated in 5% of PDAC patients [111]. Interestingly, the NCCN Practice Guidelines recommend genetic testing for ALK, NRG1, NTRK, ROS1, BRAF, BRCA1/2, HER2, KRAS, PALB2 but does not include FGFR2 [112]. However, patients with FGFR2 mutations have shown an exceptionally positive response to erdafitinib in pancreatic cancer [111,113,114]. There are two types of FGFR2 alterations: point mutation or fusion with another gene [115], and these alterations are usually found in wild-type KRAS.

According to de Almeida Carvalho, 1601 patients out of 45,750 had an FGFR mutation that could be treated with erdafitinib. This represents 3.5% of PDAC cases [116].

There are other FGFR2 inhibitors such as pemigatinib [117] and infigratinib [118,119] which have been tested in cholangiocarcinoma but not in pancreatic cancer. At the cell level, infigratinib showed important inhibition of proliferation and invasion of PDAC cell lines [120].

### 2.5. Other Frequently Mutated Genes

Other frequently found mutated genes are those involved in epigenetic control such as *ARID1A*, ARID1B, SMARCA1, MLL2, KDM6A and DNA repair genes such as BRCA2, albeit at much lower frequency [121]. These genes are not considered canonical drivers of pancreatic cancer.

Adenosquamous carcinoma of the pancreas has a similar mutation profile to PDAC [122].

## 3. Genotyping Pancreatic Cancer

Genotyping PDAC patients can have two objectives:

(1) To determine the nature of pre-invasive lesions and decide therapeutic approach;
(2) To tailor treatment to the genotype.

PDAC kills patients by two different mechanisms:

- Locally destructive cancer (30% of cases);
- Metastatic dissemination (70% of cases).

Importantly, post mortem studies found that SMAD4 mutations were very frequent in metastases and mainly absent in locally destructive PDAC [123].

Indeed, Singhi et al. [124] found that in a population of 3594 PDAC patients, genotyping permitted identifying 17% that were amenable to targeted therapies. When KRAS inhibitors are available for clinical use, this percentage should increase significantly.

## 4. KRAS Mutations

Mutated KRAS is probably one of the best-studied oncogenic mutations. Its targeting has become a top priority in pancreatic cancer treatment. There is evidence showing that KRAS mutation occurs early in PDAC development because it is frequently found in pancreatic intraepithelial neoplasia [125].

Like other members of the RAS family, KRAS is activated by the binding of GTP [126] and is inactivated by GTP hydrolysis, which is decreased or impaired in oncogenic mutant KRAS.

**Figure 2:**
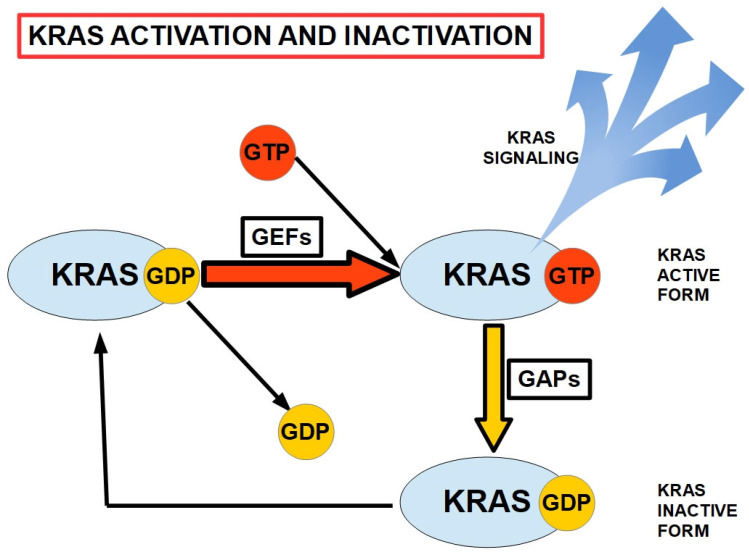
KRAS activation and inactivation. GEFs: Guanine nucleotide Exchange Factors. GAPs: GTPase-activating proteins. KRAS is activated by GTP binding [127].

**Figure 3:**
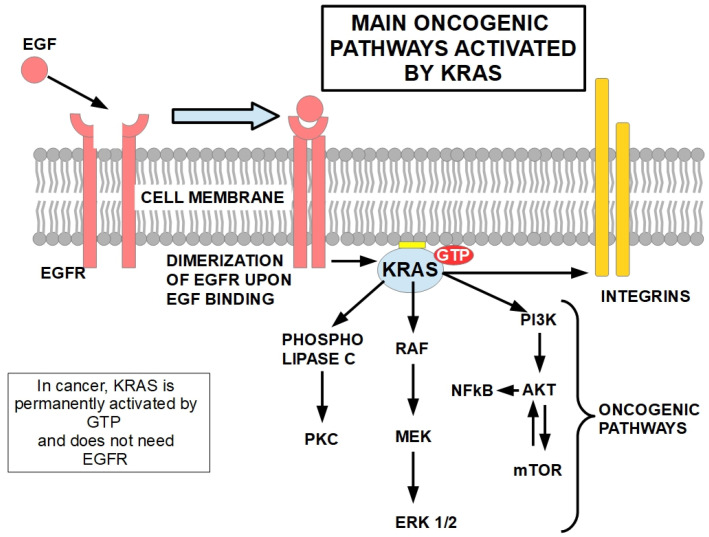
The main oncogenic pathways of KRAS activation are shown in the diagram: the MAPKinases [128,129] are on the left and the PI3K/AKT/mTOR [130] are on the right. While the MAPKinases mediate proliferative signals, the PI3K pathway enhances survival, resistance to apoptosis and synthesis of essential molecules necessary for growth and proliferation. Oncogenic mutations in KRAS stabilizes its binding with GTP, leading to the constitutive activation of its downstream signaling.

Figure 2 shows the mechanism of KRAS activation through guanine nucleotide exchange factors (GEFs), which exchange GDP for GTP. GTP-bound KRAS represents the active form of this protein that unleashes mainly pro-tumoral downstream signaling, as shown in Figure 3. Figure 2 also shows that KRAS has a low-level phosphorylase activity, which is insufficient for dephosphorylating GTP. In this regard, GTPase-activating proteins (GAPs) enhance GTP dephosphorylation. The process ends with inactive KRAS bound to GDP.

Mutated KRAS is unable to hydrolyze GTP and therefore remains activated.

The KRAS protein is the connection between cell membrane growth factor receptors and intracellular signaling networks and transcription factors of pathways related to proliferation, biosynthesis and cell survival (Figure 3). KRAS also connects with integrins involved in migration and invasion.

In Figure 3, KRAS is activated by the EGF receptor upon binding of EGF, which leads to dimerization of the receptor and autophosphorylation of EGFR C-terminal regulatory motifs with activation of downstream signaling, including KRAS.

Mutated KRAS, on the other hand, does not need EGFR activation because in this case, it remains permanently activated. Through integrins modulation, KRAS signaling controls migration and invasion [131]. Activation of phospholipase C catalyzes phosphoinositol diphosphate and generates diacylglycerol and inositol trisphosphate, which act as second messengers activating phosphokinase C and releasing intracellular calcium respectively [132]. KRAS farnesylation is essential for its attachment to the cell membrane [133] and is shown in Figure 3 as a yellow rectangle between KRAS and the inner surface of the cell membrane.

### 4.1. Types of KRAS Mutations

KRAS point mutations are usually found in codons 12 and 13 [134], and less frequently 61, which result in G12D, G12V, G12C or G13D and Q61K or Q61L mutations. G12, G13 and Q61 KRAS point mutations result in a permanently activated KRAS. The mechanism of this permanent activation is that mutated KRAS is unable to interact with GTPase-activating proteins (GAPs) that increase KRAS GTPase activity [135]. Permanent KRAS activation also results in the permanent stimulation/activation of its many downstream proteins that participate in proliferation, resistance to apoptosis/survival, and metabolic regulation.

Kameta et al. [136] obtained specimens of PDAC through endoscopic ultrasound-guided fine-needle aspiration from twenty-seven patients and found that twenty-six had KRAS point mutations of which twenty-five had G12, none had G13, and one had Q61 mutations.

### 4.2. Clinical Significance of KRAS Mutations

A meta-analysis performed on 2249 patients with pancreatic cancer and published in 17 articles showed that KRAS mutations predicted poor prognosis with lower overall survival [137]. In a retrospective analysis of 136 patients, Kim et al. [138] found that KRAS mutation patients had a worse response to gemcitabine chemotherapy and shorter overall survival compared with wild type KRAS patients. Furthermore, finding KRAS mutations in liquid biopsies and circulating DNA correlates with worse prognosis [139,140].

In seventy patients who underwent surgery with curative intent, 37 showed KRAS mutations in surgical margins that histologically were free of tumor. The group with KRAS mutation patients had significantly shorter survival time (15 versus 55 months) [95]. However, there are also publications that found no correlation between KRAS status and prognoses [141].

### 4.3. Targeting KRAS Mutations in PDAC

For many years, KRAS mutation has been considered the untargetable gene/protein [142]. The initial attempts to inhibit KRAS were through the development of farnesyltransferase inhibitors, which theoretically impede KRAS protein from binding to the cell membrane. This is a necessary step for KRAS activation. However, farnesyltransferase inhibitors produced disappointing results [143,144].

However, recent research seems to be changing the history of the untargetable KRAS. In lung cancer (non-small cell lung cancer), the common KRAS mutation is the KRAS G12C (glycine mutated into cysteine) and, importantly, two inhibitors have been developed: sotorasib [145,146] and adagrasib, both of which FDA approved [147]. Unfortunately, KRAS mutations in pancreatic cancer are KRAS G12D/V and sotorasib has no effect on them. However, the experience accumulated with sotorasib has been quite useful for the ongoing effort to develop an inhibitor of KRAS G12D/V.

While there was a clinical trial for sotorasib in KRAS G12C mutated pancreatic cancer, NCT05251038, there are no published results as the trial was withdrawn, and no further information is available. Another phase I/II clinical trial, NCT03600883 for KRAS G12C including 38 patients, had an objective response of 21% (eight patients), and progression-free survival was 4 months [148].

There is an ongoing trial for sotorasib (NCT04892017) in PDAC and other solid tumors, which is recruiting patients. There are six clinical trials with adagrasib, but they are all against tumors that hold a KRAS G12C mutation: NCT04330664, NCT06130254, NCT05634525, NCT06024174, NCT04975256, NCT03785249.

Targeting KRAS G12D has been a complex endeavor because it lacks a clear binding site near its active pocket switch 2 [149]. Therefore, this represents a challenge for binding an inhibitor molecule. Another important problem has been KRAS’s great affinity for GTP, which is abundantly distributed in the cytoplasm. Therefore, any KRAS inhibitor needs to circumvent these two factors.

Some examples of recent drugs targeting this mutant are discussed here:

#### 4.3.1. MRTX1133

MRTX1133 is an experimental drug developed on a structure-based design that specifically targets KRAS G12D and has low affinity for wild-type KRAS [150,151].

MRTX1133 showed synthetic lethality with EGFR inhibition in colorectal cancer [152] and synergistic anti-tumor effects in PDAC [153].

At the time of writing this review, there is solid preclinical evidence of MRTX1133 as an effective and potent inhibitor of KRAS G12D with benefits in PDAC, but there is no information about patient treatments [154,155,156,157]. There is one ongoing clinical trial, which is of MRTX1133 in patients with advanced solid tumors harboring a KRAS G12D mutation (NCT05737706). This phase I/II trial is still recruiting patients, and the estimated completion will occur in 2026.

#### 4.3.2. PMC79

Bras et al. [157] developed a KRAS inhibitor, PMC79, based on ruthenium that specifically targets mutated KRAS and its downstream signaling ERK and AKT proteins, without affecting non-mutated KRAS.

PMC79 targets KRAS G12D and KRAS G12V and has been experimentally tested in colorectal cancer in vitro and in vivo [158]. Although it has not been tested in pancreatic cancer with KRAS mutation, the nature of its inhibition makes this drug a very promising one. Present-day research is trying to develop more effective pan-KRAS inhibitors.

#### 4.3.3. siRNA Targeting KRAS

siRNA targeting KRAS is a mechanism that has been very useful for KRAS inhibition in the laboratory. It requires a vector that drives siRNA inside the cell. Vectors have been developed with the ability to perform this job, and the vector loaded with siRNA has been tested in association with FOLFIRINOX in a small group of patients. Overall, survival was significantly improved [159] (**NCT01188785**). However, there were only 15 patients, and there was no control group.

#### 4.3.4. Other anti-KRAS G12D trials

Table 1 shows a list of clinical trials for the treatment of PDAC with KRAS G12 mutation.

## 5. TP53 Mutation

Somatic mutations of the TP53 gene are one of the most frequently found alterations in human cancers [160]. TP53 germline mutations are infrequent and represent the cause of the hereditary cancer predisposing Li-Fraumeni syndrome (see below). The usual TP53 mutation is a point mutation due to a single-base substitution that inactivates the gene. The mainly missense mutations can affect the protein in different positions. A total of 32.6% of PDAC patients (299 TP53 mutated cases in 918 pancreatic tumors) have an inactivating TP53 mutation according to the IARC (International Agency for Research on Cancer, WHO) TP53 Database (R13, November 2008) [161]. TP53, which encodes the protein p53, is the most frequently mutated tumor suppressor gene in PDAC. Many publications report an incidence closer to 60% rather than the 32% originally stated in the literature [162].

**Figure 4:**
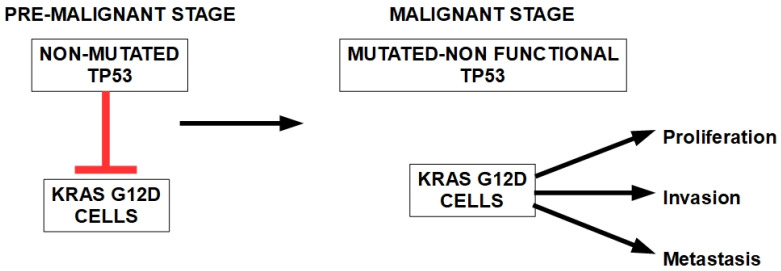
The experiment by Morton et al. [162] showed that TP53 gene and p53 protein acted as a “stop” against the expression of the malignant phenotype of KRAS transformed cells. Once TP53 is mutated with loss of function, the mutated oncogenic KRAS is fully able to develop the malignant phenotype. This is clear evidence of TP53’s tumor-suppressing abilities.

Morton et al. [163] discovered an interesting relationship between KRAS and TP53 mutation (Figure 4). They found that most of the KRAS G12D pancreatic cells (which are tumor drivers) were spontaneously and selectively lost from the pancreatic tissue. When TP53 is mutated and inactivated, the tissue retains most of the KRAS mutated cells, which quickly evolve from a pre-malignant to a PDAC stage.

Furthermore, Donehower et al. [164] showed that TP53 knockout produced spontaneous tumors in 100% of mice after 9 month and increased susceptibility to different carcinogens and ionizing radiation [165].

**Figure 5:**
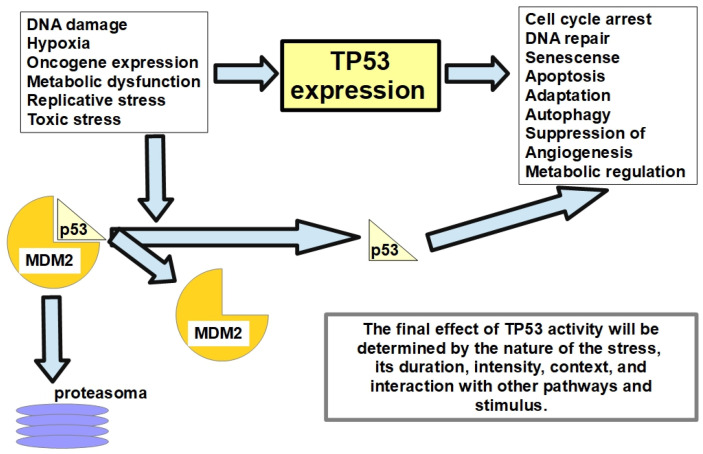
TP53 gene/protein exerts a homeostatic effect on the cell, protecting the genome, and if this cannot be preserved, eliminating the cell through apoptosis or inducing senescence. p53 protein is attached to another protein known as MDM2 (murine double minute 2), which impedes p53 activation and drives it to proteasomal destruction. p53 activation requires that it be released from MDM2 [167]. It goes beyond the scope of this paper to discuss the details and intricacies of the p53 transcription factor. For a review, see Tanaka et al. [168].

TP53’s main function consists of preserving the genome under normal and stressful conditions [166]. Figure 5 summarizes the activation and effects of the TP53 gene/protein.

## 6. BRCA Mutations

In 2005, Farmer et al. [169], Bryant et al. [170], and Helleday et al. [171] independently found that poly ADP ribose polymerase (PARP) inhibitors induced chromosomal instability, cell cycle arrest, and finally apoptosis in cells with BRCA1/2 mutations. The mechanism suggested by the authors was that “the inhibition of PARP leads to the persistence of unrepaired DNA lesions normally repaired by homologous recombination”. This finding was confirmed by many further publications also showing that PARP inhibitors could induce apoptosis in cells lacking BRCA1/2 mutations [172].

Furthermore, it was shown that PARP inhibitors were able to induce apoptosis in pancreatic cancer cells (CAPAN-1) with mutated BRCA1/2 [173]. These findings led to the development of PARP inhibitors that could be used in the clinical setting.

Targeted treatments in pancreatic cancer, such as PARP inhibitors, are limited to a small subset of patients with BRCA1/BRCA2 mutations.

**Figure 6:**
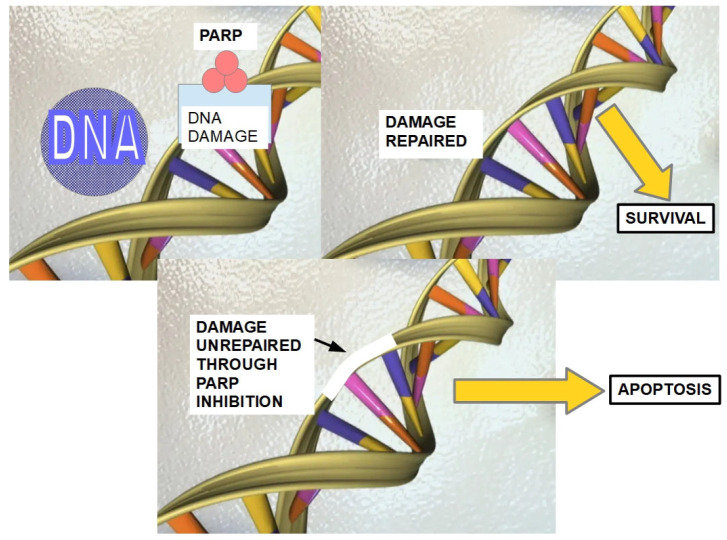
BRCA1 or BRCA2 mutations lead to difficulties in DNA repair. Inhibition of PARP enzymes further increases these difficulties. PARP enzymes are essential for single-strand break repair. Their inhibition produces a collapse of the mitotic fork, indirectly generating double-strand breaks that cannot be repaired when BRCA mutations are present, therefore inducing apoptosis [175]. This is known as synthetic lethality. In addition to Olaparib, there are other approved PARP inhibitors such as rucaparib and niraparib, which have a similar mechanism of action.

PARP1 is a sensor of DNA damage. DNA strand breaks activate PARP1, which synthesizes chains of poly (ADP-ribose) branched chains. PARP1 inhibition slows the repair of DNA damage. PARP1 inhibitors deprive the cell of the adequate mechanisms to repair single strand breaks. This collapses the mitotic fork and secondarily induces double strand breaks that should be repaired by BRCA1 and 2. When BRCA1 or BRCA2 are mutated and non-functional, double strand breaks cannot be repaired, and this damage induces apoptosis. This process is known as synthetic lethality (Figure 6). (Synthetic lethality is the situation in which the defect in one of two genes has little effect, but when both are affected, cell death results [174]).

### 6.1. Olaparib

Many PARP1 inhibitors have been developed. Olaparib was one of the first, and there is ample experience with it.

The large phase 3 POLO study found that olaparib prolonged progression-free survival compared with placebo as maintenance for patients with metastatic, platinum-sensitive pancreatic cancer and with a germline *BRCA* mutation [176]. However, although this is a breakthrough, the results show how far we are from really improving PDAC outcome: median PFS was increased from 3.8 months (placebo) to 7.4 months (olaparib).

### 6.2. Rucaparib

Rucaparib, another PARP inhibitor, showed a favorable clinical response in 4 of 19 patients with the PDAC and BRCA mutation [177], with a high number of adverse events. Better results were obtained in a phase II clinical trial with 42 patients with a 42% favorable response rate and a median response duration of 17 months [178]. Rucaparib showed improved survival in some patients with BRCA1, BRCA2, or PALB2 mutations [179].

### 6.3. Niraparib

In association with immune checkpoint inhibitors, niraparib has been tested in advanced pancreatic cancer (NCT03404960) with a stable response to platinum-based chemotherapy. Niraparib plus ipilimumab showed an improved six-month progression-free level. Importantly, patients were not selected by genetic mutation [180]. This report shows that PPAR inhibitors can have therapeutic effects beyond BRCA or PALB2 mutation and highlights the potential of PPAR inhibitors in a broader population of patients.

Patients with DNA repair defects treated with niraparib monotherapy resulted in 41% of cases of progression-free survival at 6 months [181].

While olaparib activates STAT3, niraparib inhibits this pathway. This may explain niraparib’s anti-tumoral effects in patients without defects in the DNA repair system [182].

## 7. p16 (MST1 or CDKN2A) Mutations

**Figure 7:**
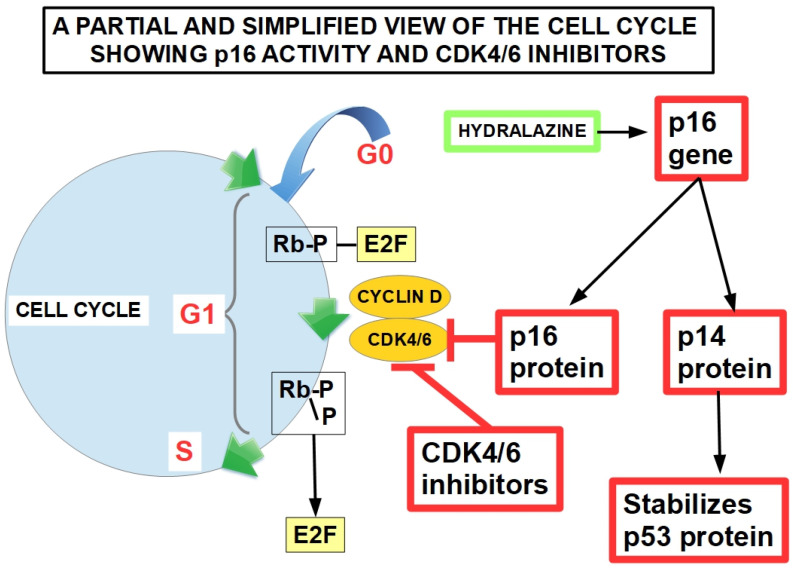
Minimally phosphorylated Rb protein is an inhibitor of the cell cycle in the G1 phase. When it is phosphorylated by the cyclinD-CDK4/6 association, it releases E2F protein and allows cell cycle continuation. Inactivation of p16, whether by mutation or epigenetic inhibition, releases CDK4/6 inhibition, which phosphorylates Rb [186]. Green arrows refer to cell cycle. Blue arrow represents the incorporation of a non-mitotic cell to the cycle.

The CDKN2A gene is a tumor suppressor gene that binds and inactivates cyclin-dependent kinase 4 and 6, thus impeding phosphorylation of various growth and regulation factors which control proliferation (Figure 7). p16 retards cell mitosis by slowing progression from the G1 to the S phase [183].

The p16 mutation permits cyclin-dependent kinase 4 activation, leading to Rb hyperphosphorylation and cell cycle progression [184].

*CDKN2A* has four exons that encode two proteins—p16 and p14ARF—through alternative splicing. p16 inhibits cyclin-dependent kinase 4, thus preventing the phosphorylation of retinoblastoma protein (Rb1). A hypo-phosphorylated Rb1 impedes mitosis by sequestering and preventing transcription factor E2F1 from inducing S-phase genes. p14ARF antagonizes MDM2, impeding ubiquitination of p53 and its proteasomal degradation [185].

The tumor suppressor p16 gene is usually silenced by deletions; however, silencing by epigenetic mechanisms has also been found in some pancreatic cancers.

### 7.1. Hydralazine

While p16 can have extensive mutations (usually deletions), this is the only driver gene in which promoter methylation has been found as a silencing mechanism in pancreatic cancer [187]. In this case, epigenetic silencing can probably be targeted with hydralazine, which has shown the ability to re-express p16 [188,189,190,191,192]. However, this drug has not been tested in pancreatic cancer, and we did not find any publication on the rate of p16 epigenomic silencing versus deletion in pancreatic cancer. Interestingly, hydralazine was found to have three important characteristics that may influence PDAC treatment:

- Hydralazine increases nanoparticle drug penetration in desmoplastic tumors [193];
- Hydralazine inhibits glutamate oxaloacetate transaminase-1 (GOT1), thus impeding abnormal glutamine metabolism in various tumor cells, including pancreatic cancer cells [194]. Importantly, inhibition of GOT1 can promote pancreatic cancer cell ferroptosis [195]; it was found that KRAS-mutated cancer cells rely heavily on GOT1 for long-term proliferation, and GOT1 inhibition sensitizes the malignant cells to glucose deprivation [196].
- On the negative side, while hydralazine was able to revert gemcitabine resistance in cervical cancer cells [197], it failed to do so in pancreatic cancer cells [198].

### 7.2. CDK (Cyclin Dependent Kinases) Inhibitors

Although there are no clinically available methods to restore p16 function when it is silenced through deletions, a proxy would be to inhibit downstream effectors such as CDK4/6. Importantly, CDK4/6 inhibitors have been developed and are in clinical use in tumors such as breast cancer [199]. There are three FDA-approved CDK4/6 inhibitors for breast cancer: palbociclib, ribociclib, and abemaciclib. However, the toxicity of these oral drugs is high, and their therapeutic value is under discussion. The Pallas and Penelope B studies with palbociclib failed to meet their primary endpoints. In the case of breast cancer, CDK inhibitors were tested without genotyping for p16 mutations. Theoretically, pancreatic cancer with p16 loss of function mutations should be more responsive to CDK inhibitors. But theory and practice frequently fail to coincide. Response to these inhibitors in PDAC was very variable in pancreatic cancer models [199]. Furthermore, some chemotherapeutic drugs may be antagonized by these inhibitors. That is the case of taxanes. On the other hand, mTOR and MEK inhibitors showed synergism with CDK4/6 inhibitors. Some PDAC cells have a strong growth-inhibiting response to CDK4/6 inhibition, e.g., CAPAN2 cells, while others were fully resistant, e.g., HS766T, ASPC1, and PL5 [200].

CDK inhibitors have shown therapeutic effects in PDAC, but paradoxically, independently from p16 mutation. For example, Dinaciclib [201] is a multi-CDK inhibitor that does not act against CDK4/6, and it has shown efficacy in pancreatic cancer in vitro and in vivo mouse model systems [202]. Something similar was found with CDK1 inhibitors [203].

Two clinical trials have failed to show overall survival improvement:

NCT02981342: A Study of Abemaciclib (LY2835219) Alone or in Combination With Other Agents in Participants With Previously Treated Pancreatic Ductal Adenocarcinoma.

NCT02985125: LEE011 Plus Everolimus in Patients With Metastatic Pancreatic Adenocarcinoma Refractory to Chemotherapy.

The conclusion is that stand-alone CDK4/6 inhibitors in p16-mutated or -silenced tumors have low therapeutic effects, but they can be improved with additional inhibition of MEK, ERK, or mTOR inhibitors [204].

## 8. SMAD4 Mutations

**Figure 8:**
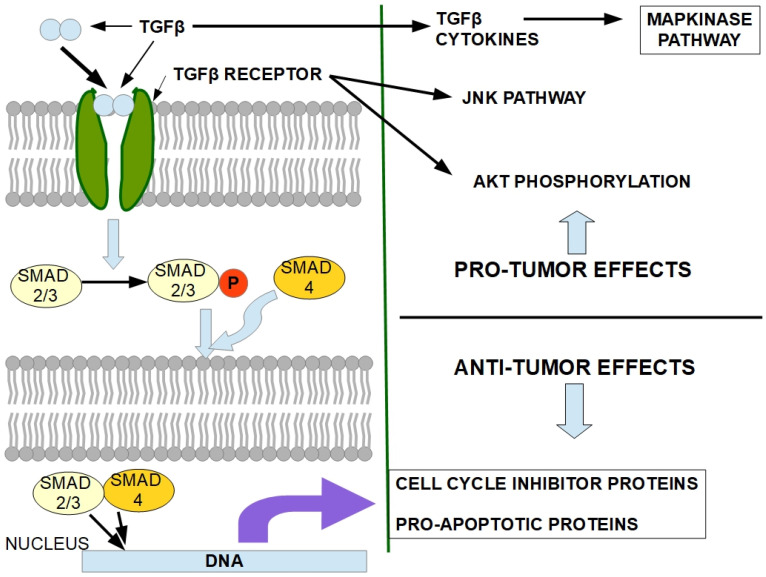
(**Left panel**) The SMAD pathway. Upper right panel: Pro-tumoral effects of TGF-β. (**Lower right panel**) Anti-tumoral effects of the SMAD-TGF-β pathway. Purple arrow represents transcription. Light blue arrows mean effects.

SMAD4 is a member of the TGF-β pathway acting as a tumor suppressor. SMAD4 mutations disrupt TGF-β pathway signaling [205]. While SMAD4 loss is not an initiator of pancreatic cancer, it can promote tumor progression usually initiated by KRAS activation [206]. TGF-β is a dual-acting protein with protumoral and anti-tumoral actions. While SMAD4 is not mutated and is functionally active, the TGF-β-SMAD4 pathway is clearly anti-tumoral by promoting the transcription of cell cycle inhibitors such as p15, p21, and p27 that block cyclin dependent kinases and induce growth arrest [207,208] and apoptosis by inducing the transcription of pro-apoptotic genes [209]. When SMAD4 is functionally lost by mutation or epigenetic blocking, the pro-tumoral activity of TGF-β is fully evident [210] (Figure 8).

Unfortunately, as yet there is no way to reestablish the expression of the lost SMAD4. However, a possible proxy to this would be inhibition of TGF-β. Antibodies have been used in this regard by interfering with TGF-β ligand binding to the receptor. Inhibition of TGF-β signaling is a two-way street because of its dual activity: pro- and anti-tumor effects. Fresolimumab, an antibody that binds TGF-β, was tested against melanoma and renal cancer [211] and breast cancer [212]. Results were modest, and diverse skin tumors developed in some patients [213]. It has never been tested in pancreatic cancer patients. However, fresolimumab has one important effect that may be advantageous in pancreatic cancer: it inhibits fibrosis, which is known to be a major contributor to PDAC progression and poor prognosis [214,215,216,217].

## 9. Familial Pancreatic Cancer (FPC)

In 1973, MacDermott and Kramer described a family in which four out of six siblings developed pancreatic cancer [218]. Since then, a great number of families with clustering of PC were reported [219,220,221,222,223,224]. A specific gene that explains familial PC has not been identified beyond doubt. However, there is a group of genes whose mutation may participate in FPC: BRCA2, p16, STK11 [225].

Individuals that belong to families in which two or more of these mutations occur were identified have a higher risk for pancreatic cancer [226,227,228,229,230,231,232,233,234]. There is evidence that these cases have a genetic base [235]. The PALB2 gene was discovered to be a familial PC susceptibility gene [236]. This gene is a binding partner of BRCA2. Mutations of BRCA2 and PALB2 are the most frequently found genetic alterations in FPC. Independently of the FPC genotype, efforts should be directed towards screening for early detection.

## 10. Hereditary Cancer Predisposition Syndromes

In addition to familial pancreatic cancer, there is a group of hereditary mutations that predisposes for cancer in general and for certain types of cancer in particular. Some of these diseases also predispose for pancreatic cancer.

### 10.1. Hereditary BRCA1 or BRCA2 Mutation

This pathology increases the risk for breast, ovarian, and prostate cancers. Pancreatic cancer risk is also increased, with a relative risk in the range of 2–2.5 [237]. The mechanism involved is increased genomic instability through impaired DNA repair. BRCA mutations per se do not produce pancreatic cancer until other somatic oncogenic mutations occur.

### 10.2. Li-Fraumeni Syndrome

This syndrome is characterized by inactivating mutations of TP53 [238]. Detection and apoptosis of genetically altered cells is decreased; thus, the risk of mutations in other genes is increased. Sarcomas, leukemia, breast, brain and pancreatic cancer incidence is increased in these patients. As in the case of BRCA, TP53 mutation does not induce pancreatic cancer until other somatic oncogenic mutations take place.

### 10.3. Lynch Syndrome (Hereditary Non-Polyposis Colorectal Cancer)

Several DNA repair enzymes such as MLH1, MSH2, MSH6, PMS2, EPCAM can be mutated in this syndrome. This creates a genomic instability that increases the risk of malignant tumors in the colon, rectum, stomach, small intestine, gall bladder, brain, prostate, upper urinary tract, and pancreas. The relative risk for pancreatic cancer is low (RR 1.31) under age 50, but then increases significantly at age 70 (RR 3.68) [239,240].

As in the above-mentioned hereditary syndromes, the development of pancreatic cancer requires the somatic mutation of other oncogenic genes, which occurs with a higher frequency than in the normal population.

### 10.4. Familial Atypical Multiple Mole Melanoma (FAMMM)

This syndrome has an increased incidence of malignant melanomas and moles. It is the consequence of a germline mutation of CDKN2A(p16) or CDK4 [241]. The most frequently found genetic lesion is a 19-base pair deletion known as p16-*Leiden* that extends from nucleotide 225 to 243 on chromosome 9 [242].

These patients are prone to develop pancreatic cancer as well [243]. Both types of altered genotype, CDKN2A and CDK4, have a higher risk for pancreatic cancer. The relative risk is increased 13- to 38-fold [244]. The mechanism of action is through the loss of mitotic restriction by CDKN2A or the pro-mitotic activity of CDK4.

### 10.5. Peutz-Jeghers Syndrome

Peutz-Jeghers syndrome is the consequence of germline mutation with loss of heterocigocity of the tumor repressor gene STK1/LKB1 (autosomal dominant inheritance with complete penetrance) [245]. Hamartomas and malignancies of the digestive system are increased.

One out of every four persons with the Peutz-Jeghers syndrome at age 70 has developed pancreatic cancer at an average age of 55 [246]. While the TP53 mutation in the Li Fraumeni syndrome showed a 7.7 relative risk for pancreatic cancer [247], Peutz-Jeghers syndrome has the highest cumulative rate of pancreatic cancer when compared with the other hereditary cancer predisposition syndromes (33%) [248]. Interestingly, it is unusual to find mutations of SMAD4, TP53, and KRAS, and it is probable that the STK1/LKB1 silencing follows a different mechanism from that found in sporadic cases to produce pancreatic cancer [249]. One possible pathway leading to cancer is the loss of LKB1 “master-kinase” function, which modulates cellular processes such as cell polarity, energy metabolism, and cell growth. LKB1 regulates these processes partly via the AMP-activated protein kinase/mammalian target of rapamycin (AMPK/mTOR) signaling pathway. Inactivation of LKB1 results in up-regulation of mTOR activity [250].

### 10.6. Multiple Endocrine Neoplasia Type 1

Multiple endocrine neoplasias type 1 is produced by mutation of the MEN1 gene. This syndrome is characterized by parathyroid and pituitary tumors. Regarding the pancreas, it increases the risk of pancreatic endocrine tumors, but this too is beyond the scope of this review.

### 10.7. Von Hippel Lindau Disease

Von Hippel Lindau disease is produced by an inactivating mutation of VHL. Von Hippel-Lindau disease usually produces hemangioblastomas, clear cell renal carcinoma, neuroendocrine tumors, pheochromocytoma, and pancreatic anomalies, mainly cysts (15% incidence), but these usually do not progress towards pancreatic malignancy. Endocrine pancreatic tumors are also frequent [251,252].

## 11. Gene Signatures and Prognosis of Pancreatic Cancer

There are many publications showing gene signatures with better and worse prognoses for pancreatic cancer [253,254,255,256]. The prognostic value of these signatures is very relative because even those tumors with supposedly “better” prognoses make a very small difference regarding overall survival. On the other hand, the situation may be different in operable tumors where better overall survival can be expected. Wu et al. [257] identified five genes useful for this purpose: AADAC, DEF8, HIST1H1C, MET, and CHFR. Huang et al. [258] identified another four genes that supposedly allow relapse risk measurement (DNAH9, TUBGCP6, and TMEM132E).

## 12. Genetic Interactions in PDAC

In a mouse model of PDAC, Aguirre et al. [259] showed that KRAS G12D mutation alone produced focal premalignant ductal lesions, such as pancreatic intraepithelial neoplasias. CDK2N (p16) inactivation alone had no effect at all in the pancreas. But, when both mutations were present, pre-malignant lesions evolved swiftly to highly invasive and metastatic cancers. Interestingly, this genetic mutational association had a very important stromal proliferative effect that was absent in individual mutations. According to the authors, activated KRAS serves as the initiator of the premalignant lesion, while the p16 tumor suppressor loss determines the malignant conversion into invasive ductal adenocarcinoma.

On a speculative basis, we may assume that TP53 and BRCA mutations play an important role in genetic instability and clonal progression. SMAD4 inactivation seems to be an important contributor to the desmoplastic reaction of PDAC [260,261], migration, and metastatic behavior.

## 13. Genetic Profile of Cells Obtained by Liquid Biopsy (LB) or from Secretions in Pancreatic Cancer

Tumors can release cells and parts of cells that can then be found in blood and other biological fluids. These findings are becoming useful for diagnostic and prognostic purposes. The released materials are circulating tumor cells (CTCs), circulating cell free nucleic acids (cfDNAs and cfRNAs), and circulating extracellular vesicles, e.g., exosomes.

cfDNAs reach the blood stream through cell turnover or cell death [262]. While cfDNAs are increased in PDAC [263,264], they have not, as yet, been systematically introduced in clinical practice in pancreatic cancer. The analysis of these materials requires sequencing or other molecular methods to identify specific mutations that can be found in pancreatic cancer. These mutations usually are in the *KRAS*, *CDKN2A*, *TP53* and *SMAD4* genes. However, these mutations can also be found in non-malignant diseases or low-grade intraductal neoplasias [265].

### 13.1. Circulating Tumor Cells (CTC)

Kulemann et al. [266] studied CTC specimens from 58 patients with PDAC in which 67% of patients showed CTCs either as clusters or single cells. The remaining patients were negative for CTCs. In the patients with CTCs, 72% had a KRAS mutation in the CTC, and 48% had both positive CTC and KRAS mutation. Patients with a higher tumor burden in the blood (>3 CTC/mL) showed a trend to poorer OS (11.5 months vs. 20 months). The CTC and the primary tumors had equally-distributed genotypes of KRAS mutations, but CTC showed more diverse KRAS mutations. Surprisingly, the KRASG12V mutation in CTC was associated with better OS (median OS 24.5 months) compared to KRASwt.

It is of note that of the 21 patients with KRAS mutations in the CTC and the primary tumor, only 58% had a matching mutation in CTC and tissue. The other 42% had discordant mutations.

### 13.2. Pancreatic Exocrine Secretions

A meta-analysis of 2156 patients concluded that detection of KRAS mutations in pancreatic exocrine secretions did not provide sufficient specificity or sensitivity to distinguish PDAC patients from chronic pancreatitis, or pre-malignant lesions, or healthy individuals [267].

### 13.3. Circulating DNA

Promoter methylation of genes is frequently found in circulating DNA. They may represent a good tool for early diagnosis of pancreatic cancer. The following genes have been found in cfDNA: ADAMTS1 and BNC1 [268], SPARC (Secreted Protein Acidic and Rich in Cysteine), UCHL1 (ubiquitin carboxy-terminal hydrolase L1), PENK (proenkephalin), and NPTX2 (neuronal pentraxin 2) [269]. Accumulating evidence shows that the methylation profiles of certain genes in cfDNA are a useful marker for distinguishing between chronic pancreatitis and cancer [270], and some like SPARC may be an early marker of the disease.

### 13.4. Circulating Exosomes

The importance of exosomes for diagnostic purposes lies in their content, which represents the message they are carrying. Two miRNAs, -21 and -221, are particularly over-expressed in pancreatic cancer tissues [271]. Down-regulation of these two miRNAs decreased migration/invasion and reduced the expression of NF-kB and KRAS. Exosomal miRNA 21 is usually found to be increased in PDAC with a sensitivity of 95.5% and specificity of 81.5% [272].

An important diagnostic finding in exosomes originating from pancreatic cancer cells is the expression of glypican1 [273]. This membrane-anchored protein is expressed in PDAC with great specificity and sensitivity, and importantly, in an early stage of the disease.

## 14. DNA Methylation in Pancreatic Cancer

DNA methylation/demethylation is a mechanism that permits epigenetic control of transcription and genome structure. Aberrant patterns of methylation/demethylation are strongly associated with carcinogenesis and gene transcription, in tumors including pancreatic cancer [274].

In 2003, Sato et al. [275] identified seven over-expressed genes in pancreatic cancer that were due to hypomethylation. These were *CLDN4* (claudin 4), *LCN2 (lipocalin 2)*, *MSLN* (mesothelin), *PSCA* (prostate stem cell antigen), *S100A4*, *SFN* (stratifin) and *TFF2* (trefoil factor 2). Table 2 shows the major effects of each of these genes.

Tan et al. [291] have shown that aberrant gene methylation is a frequent finding in pancreatic cancer. They identified 23 genes that were regulated by hypermethylation and 35 by hypomethylation. Mishra et al. [292] found 23,000 CpG island methylated differently in pancreatic cancer compared with normal pancreatic cells. This gives an idea of the complexity of the issue. Furthermore, Zhu et al. [293] identified 45 CpG island that, when methylated, increased or decreased the risk of pancreatic cancer. Studying the possible target genes of this aberrant methylation led to the identification of six genes that can be related with cancer risk, namely *ABO*, RPS2, SURF6, ORMDL3, SNHG9 and *SOWAHC.*

The ABO gene determines the blood group, and it is known that those with a non-O blood type have a higher risk for pancreatic cancer [294]. SURF6 methylation, which has been identified as a risk factor in breast cancer [295], is now also associated with pancreatic cancer. ORMDL3 has been associated with increased expression in the bronchial tree in smokers [296] and causes endoplasmic reticulum stress [297]. On a speculative basis, we may assume that ORMDL3 demethylation represents the unidentified link between cigarette smoking and increased risk of pancreatic cancer [298]. SNHG9 codes for a lncRNA that is a prognostic marker of pancreatic cancer [299]. It was shown in lung cancer that SNHG9 can downregulate miR-21 through methylation, thus suppressing malignant cell proliferation [300]. SNHG9 has also shown pro-tumoral effects in some tumors, such as hepatocellular carcinoma by increasing proliferation, migration and invasion [301], and glioblastoma by promoting aerobic glycolysis and proliferation [302]. *SOWAHC* expression seems to be related with the immunosuppressive environment that characterizes pancreatic cancers [303].

## 15. Discussion

The key mutated gene in PDAC is KRAS. It is the initiator and an important player in disease progression. It is the number one gene whose expression increases proliferation, resistance to apoptosis, survival of the cancer cells, EMT (epithelial mesenchymal transition), biosynthesis, glycolysis, pentose phosphate pathway, reduction of ROS, increased autophagia, and micropinocytosis. To achieve this wide scope of pro-tumoral effects, it needs also the elimination of some tumor suppressor genes’ activity, such as p16, TP53 and SMAD4. BRCA1/2 mutation is probably not an essential event for KRAS to unleash its tumoral effects.

Therefore, mutated KRAS inhibition/silencing should be considered as the main step towards a more rational treatment of PDAC. This was not possible up until a few years ago. Presently, we have at least two candidate drugs that may represent the first serious breakthrough for PDAC treatment: MRTX1133 and PMC79.

Mutated tumor suppressors cannot be restored to activity with the available clinical technology. It is probable that this will change in the future. However, there are mechanisms that can partially compensate for this shortcoming. For example, if p16 is epigenetically silenced, hydralazine can re-express it. This would not work if it is mutated.

Once it is mutated, SMAD4 is definitively lost. However, the TFG-β/SMAD4 pathway can be modulated through TFG-β inhibitors that are undergoing accelerated development. Another proxy mechanism to circumvent an SMAD4-inactivating mutation is the inhibition of the MAPKinase pathway [304]. The situation with the BRCA1/2 mutation is different from the other tumor suppressor genes because this sensitizes malignant cells to PARP1 inhibitors.

In lung cancer targeting, the KRAS G12C mutation has been shown to have add-on effects/synergy with other treatments such as mTOR and IGF1R inhibitors in vitro and in vivo [305,306]. Although there is no evidence to support that similar effects can be achieved in PDAC having other KRAS mutations, the issue deserves to be investigated.

### EGFR

While EGFR-activating mutations have been found in less than 4% of PDAC patients [307,308], EGFR amplification is found in approximately 40% of patients [309] but is not usually considered to be a driver of PDAC. However, there is evidence that EGFR inhibition can improve overall survival [310,311,312,313]. EGFR mutation has been found to induce synthetic lethality with KRAS mutation in lung adenocarcinoma [314], which could explain why both mutations do not usually coexist in pancreatic cancer.

On a speculative approach, we may suppose that in non-mutated (wild type) KRAS PDAC, EGFR amplification or mutation can be a tumor driver and, therefore, requires a different targeted approach. In this regard, erlotinib has been used as an EGFR tyrosine kinase inhibitor. In 2005, the FDA approved erlotinib in combination with gemcitabine as a first-line treatment in locally advanced and metastatic PDAC [315]. It made no distinctions between KRAS conditions.

However, critical voices have been raised against erlotinib, arguing that the improvement in overall survival was scarce and the decrease in quality of life was significant, while cost-effectiveness was negative [316]. There is evidence that associating a MEK inhibitor further enhanced the erlotinib’s inhibitory activity [317]. Interestingly, most studies with erlotinib in pancreatic cancer do not distinguish between mutated KRAS and wild-type KRAS populations. On a speculative basis, we may suppose that in mutated KRAS patients, the benefits of erlotinib would be minimal. On the other hand, in wild-type KRAS, EGFR may be the driver gene, and the response to erlotinib should improve.

Use of genotyping PDAC Tumors

Less-developed countries and few developed countries do not routinely genotype pancreatic tumors. Two reasons are behind this conduct: either a lack of resources or a nihilistic approach that will defeat our best therapeutic intentions, no matter what we do.

In the second case, a mentality change is necessary because:

i. KRAS inhibition at the clinical level is a short step away;
ii. BRAF V600E-driven tumors can be successfully treated with available medications (see Table 2).
iii. BRCA 1/2 mutation offers a small but definite opportunity to improve therapeutic outcomes.
iv. KRAS G12C mutation has specific medication.
v. EGFR amplification can be approached with EGFR inhibitors that are clinically available.
vi. p16 methylation without mutation can be re-expressed with hydralazine.
vii. CDK inhibitors will probably be useful in p16 mutated tumors, but not as stand-alone drugs, even if associated with MEK inhibitors and/or mTOR inhibitors.
viii. STK1/LKB1 mutated tumors probably represent a completely different line of PDAC that has not been adequately investigated as yet. It is possible that these tumors require a different therapeutic approach.

Slowly but steadily, a personalized treatment profile on the genetic makeup of PDAC is starting to develop. We summarize the therapeutic options available according to tumor genotype in Table 3.

**Figure 9:**
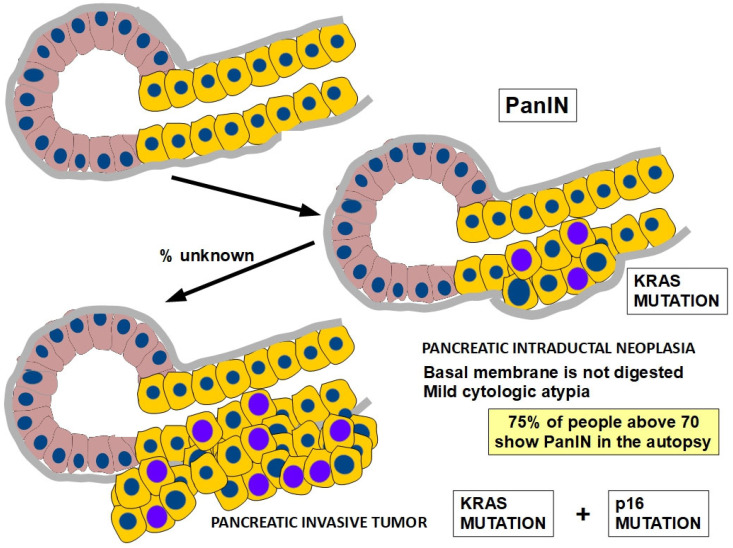
The series of mutations that drive locally invasive PDAC. See text for details.

**Figure 10:**
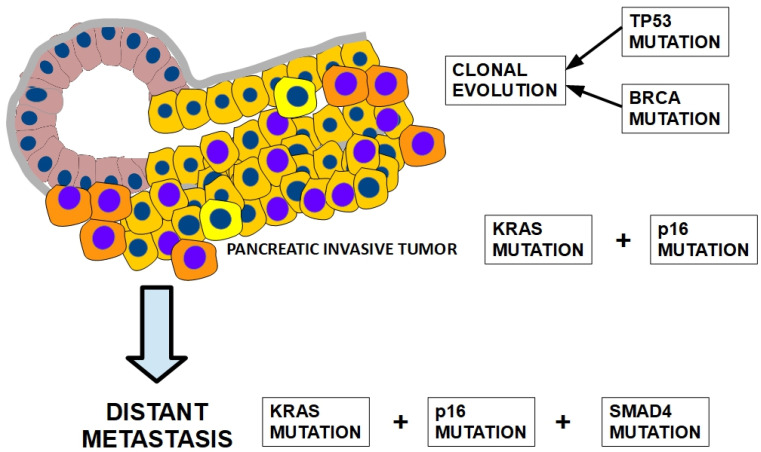
Distant metastasis can occur earlier when there is a SMAD4 mutation. See text for details.

In conclusion, Figure 9 and Figure 10 illustrate the most frequently found chronology of mutations involved in locally invasive and metastatic PDAC evolution, respectively. This chronology does not claim to be a rule because pancreatic cancer progression does not evolve in the orderly fashion usually found in colon cancer. Only driver genes are shown in the figures.

Evidence-based alternative treatments

Some alternative treatments, mostly originating in plants, have been identified, and they may have effects on the altered genotype of pancreatic cancer. Information is scanty and has not been fully investigated.

Nutlin analogs (MI-219 and MI-319) have shown an ability to inhibit MDM2, thus enhancing p53 expression in pancreatic cancer [322]; however, this will not occur in tumors with mutated p53. Considering that only 50% of patients have p53-inactivating mutations, the other 50% of are potential candidates for MDM2 inhibition.

Fucoidan, a sulfated polysaccharide from marine algae, specifically brown seaweed, has been shown to interfere with NF-kB-p53 crosstalk, increasing p53 expression and inducing apoptosis in pancreatic cancer cells [323] and other tumors [324,325]. However, fucoidan’s pharmacologic effects are so diverse that it is difficult to establish its effects on p53 [326]. Interestingly, fucoidan has shown CDK4/6 inhibitory abilities in urothelial cells [327]. In lung cancer cells, fucoidan can modulate the expression of p53 and p21 in addition to pro-apoptotic proteins [328]. For a review on fucoidan, see Hsu and Hwang [329].

## 16. Future Perspectives

If the ongoing clinical trial with MRTX1133 shows benefits similar to those found in the preclinical setting, this drug will become the first important breakthrough in pancreatic cancer chemotherapy.

The accelerated development of nanoparticles that can deliver drugs and siRNA into the malignant cells may also change the poor outlook for PDAC.

Antibody drug conjugates (ADCs) may also represent another source of new treatments. Many PDAC cells over-express nectin-4 on the cell surface, independently of their genomic profile [330]. Enfortumab vedotin is an ADC that targets nectin-4 expressing tumors and clinical trial NCT05915351 is exploring its performance.

Exosomes and circulating cancer cells show genetic signatures that will permit an earlier diagnosis of pancreatic cancers.

These new developments further emphasize the importance of genotyping pancreatic exocrine tumors.

## 17. Conclusions

An integral treatment of PDAC makes it necessary to genotype the tumor with the objective of targeting the involved genes. Conventional chemotherapeutic protocols have been shown to be unable to significantly improve survival in non-surgical PDAC. Personalized PDAC treatment is possible within a limited scope, since a few new pharmaceuticals have been developed. Mutated KRAS and wild-type KRAS tumors should be regarded as two different types of tumors. While the first group would benefit from KRAS inhibitors, the second group would probably benefit from EGFR inhibitors. STK1/LKB1 mutated pancreatic cancers seem to represent a different genetic mechanism.

## Figures and Tables

**Table 1:** List of clinical trials for the treatment of PDAC with KRAS G12 mutation.

NCT	Treatment
NCT03745326	White blood cells from the patient, grown in the lab, genetically modified, then given back to the patient: gene transfer using anti-KRASG12D mTCR cells.
NCT06218914	Phase I Study of NT-112, an autologous T-cell therapy product genetically engineered to express an HLA-C*08:02-restricted T cell receptor (TCR), targeting KRASG12D mutant solid tumors.
NCT04853017	Phase I study of ELI-002 immunotherapy. This is a lipid-conjugated immune stimulatory oligonucleotide plus a mixture of lipid-conjugated peptide-based antigens containing peptides for G12D, G12R, G12V, G12C, G12A, G12S and G13D.
NCT06040541	Phase I study of RMC-9805 in Kras G12D mutated PDAC patients.
NCT05726864	Phase I/II study of ELI-002
NCT03608631	Phase I trial study of mesenchymal stromal cells derived exosomes with KrasG12D siRNA (iExosomes) in treating pancreatic cancer with KrasG12D mutation
NCT06179160	Phase I study of INCB161734 in KRAS12G12D mutated pancreatic cancer
NCT05846516	Phase I study of the vaccine ATP150/ATP152 and VSV-GP154 treatments with and without the immune checkpoint inhibitor ezabenlimab
NCT06208124	Phase I study of the RAS inhibitor IMM-6-415 in solid tumors.
NCT05585320	Phase I study of IMM-1-104 against RAS mutated solid tumors

**Table 2:** Pro-tumoral effects developed by hypomethylated genes.

Gene/Protein	Effects	References
*CLDN4*	Through the formation of tight junctions (TJ) impedes drug penetration. Non TJ claudin 4 increases proliferation, epithelial mesenchymal transition, and stemness. Targeting claudin 4 in PDAC increases chemosensitivity.	[276,277,278]
*LCN2*	Lipocalin is a secreted glycoprotein that induces proliferation, angiogenesis, invasion (through modulation of metalloprotease 9) and metastasis.	[279]
*MSLN*	Mesothelin is a glycosylphosphatidylinositol bound to the external surface of the cell membrane. It induces the NF-kB pathway, interacts with MUC18 favoring implantation of peritoneal metastasis, and plays a role in drug resistance.	[280]
*PSCA*	Is a membrane surface antigen correlated with advanced stages and tumor progression. It is also over-expressed in metastasis. Blocking PSCA protein with antibodies has a lethal effect in pancreatic cancer cells.	[281,282]
*S100A4*	Is a small calcium binding protein that increases tumor progression and promotes metastasis (is a specific metastasis-related protein). It also inhibits apoptosis and has a role in chemoresistance.	[283,284,285,286]
*SFN*	Is a cell cycle checkpoint protein involved in tumor progression. It is usually co-expressed with cofilin 1. SFN over-expression is considered a marker of poor prognosis.	[287]
*TFF2*	Induces pancreatic cancer cells (PANC1) migration. TFF2 impedes migration and maturation of dendritic cells in PDAC. However, it also shows anti-tumoral effects.	[288,289,290]

**Table 3:** Drugs that can modify the effects of the genetic signature of PDAC.

Driver Gene or Genetic Mutations	Direct Inhibitors	Drugs Acting Indirectly
MUTATED KRAS	MRTX1133	HYDRALAZINE
	PMC79	FGTI-2734
KRAS G12C mutation	Sotorasib Adagrasib	
BRCA1/2 PALB2 other DNA repair genes		PARP1 INHIBITORS olaparib, rucaparib, niraparib
p16		HYDRALAZINE CDK inhibitors
SMAD4		TGF-β INHIBITORS MEK inhibitors
EGFR amplification and/or activating mutations	ERLOTINIB and other EGFR inhibitors	
BRAF V600E mutation and other BRAF mutations	BRAF inhibitors such as dabrafenib [318,319], and vemurafenib [320]	Trametinib
FGFR2	Erdafitinib	

Trametinib is an inhibitor of MEK1 and MEK2 that has been approved for use in combination with dabrafenib for unresectable or metastatic tumors with a BRAF V600E mutation. FGTI-2734 is a dual farnesyltransferase and geranylgeranyltransferase inhibitor under experimentation [321].

## Data Availability

Not applicable.
